# 

**Published:** 1857-04

**Authors:** 


					No. IX.]
MARCH.
as
lSCttDIKG THE
jrejt AND tECTCKRP.
w
? HK tMfZ
V
?? irpeefii'trrrf ftaicttpiv^
. THE ;
)MAS
1857.

				

